# Endoscopic Endonasal Dacryocystorhinostomy Combined with Canaliculus Repair for the Management of Dacryocystitis with Canalicular Obstruction

**DOI:** 10.1155/2015/657909

**Published:** 2015-04-08

**Authors:** Yunhai Tu, Zhenbin Qian, Jiao Zhang, Wencan Wu, Tianlin Xiao

**Affiliations:** ^1^Eye Hospital at Wenzhou, Wenzhou Medical University, 270 West Xueyuan Road, Wenzhou, Zhejiang 325027, China; ^2^Eye Hospital at Hangzhou, Wenzhou Medical University, 618 East Fengqi Road, Hangzhou, Zhejiang 310020, China

## Abstract

*Purpose*. The aim of this study is to propose a simple and efficient combination surgery for the management of dacryocystitis with canalicular obstruction. *Methods*. A retrospective noncomparative case series of dacryocystitis with canalicular obstruction has been studied. Twelve patients with dacryocystitis and canalicular obstruction underwent a conventional endoscopic endonasal dacryocystorhinostomy (EE-DCR) combined with a modified canalicular repair. Postoperative observations included slit lamp, fluorescein dye disappearance test, lacrimal syringing, lacrimal endoscopy, and nasal endoscopy. *Results*. After 6–18 months of postoperative follow-up, the symptoms of epiphora and mucopurulent discharge disappeared completely in 10 patients, and occasional or intermittent epiphora remained in 2 patients. All of the twelve patients showed an opened intranasal ostium and normal fluorescein dye disappearance test. Patent bicanalicular irrigation was achieved in 9 patients. One patient had a partial and the other two had a complete reobstruction by lacrimal irrigation to their repaired lower canaliculus; however, all of them had a patent lacrimal irrigation to upper canaliculus. The functional success rate for the combination surgery is 83% (10/12), and anatomical success rate is 75% (9/12). *Conclusion*. EE-DCR combined with modified canalicular repair is a simple and efficient method for the management of dacryocystitis with canalicular obstruction.

## 1. Introduction

Dacryocystitis combined with canalicular obstruction is not common but difficult to manage [1, 2]. Epiphora with mucopurulent discharge is the major presenting compliant; however, both of nasolacrimal duct and upper or lower canalicular duct show a blockage when probing or dacryocystography is performed. Conventional management of dacryocystitis with canalicular obstruction includes external dacryocystorhinostomy (Ex-DCR) or endoscopic endonasal dacryocystorhinostomy (EE-DCR) combined with laser or intubation or both [3–5]. Those combination surgeries are not difficult to perform, but some special equipment like laser is needed, or the results may not be satisfied [5–7]. Restenosis or scar formation probably is correlated with the unsatisfied outcomes. Finding a simple and efficient combination surgery to manage the complicated disorder is critical. Following report is a surgical technique recently we successfully used in the patients with dacryocystitis and canalicular duct obstruction. To the best of our knowledge, this is the first report about using EE-DCR combined with canalicular repair to manage dacryocystitis and canalicular obstruction.

## 2. Patients and Methods

A retrospective noncomparative study of EE-DCR combined with canalicular repair was performed from April 2013 to July 2014 in the Affiliated Eye Hospital of Wenzhou Medical College. This study was approved by the Institutional Ethics Committee, and informed consent was obtained from all the patients. Twelve consecutive patients with dacryocystitis and lower canalicular obstruction were included. The twelve patients are female, aged from 31 to 77 years with an average age of 50 years. Epiphora and mucopurulent discharge are their major complaints, with an average duration of 7 years. Among twelve patients with dacryocystitis, four were bilateral and the remaining eight were unilateral. As to the twelve patients with canalicular obstruction, nine were left lower canalicular obstruction, and three were right lower canalicular obstruction. The distinguishing features of this disorder included the following: mucopurulent discharge was refluxed from upper canalicular punctum when irrigated with saline; a probe could not be passed through or touched to the lacrimal bone when to probe the obstructed lower canaliculus; CT dacryocystography showed an enlarged lacrimal sac. The general information of twelve patients as well as the site of lacrimal canalicular obstruction (distance from punctum to canalicular obstruction site) is summarized in Table 1.

### 2.1. Surgical Procedure

**Figure 1:**
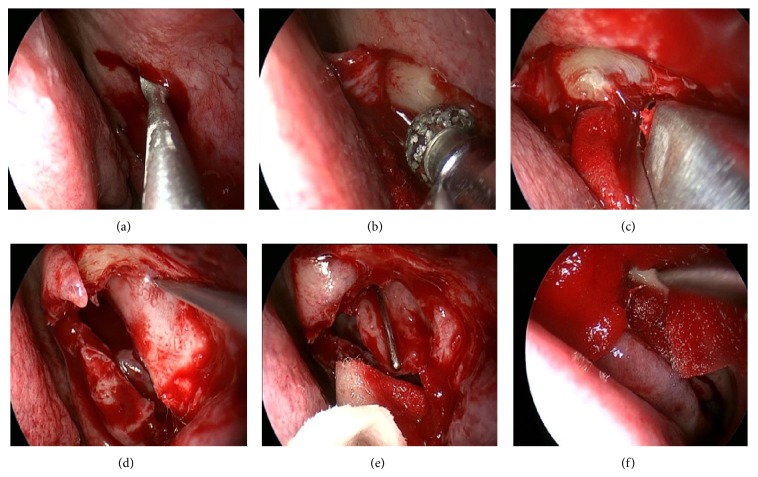
EE-DCR. A square nasal mucosal flap was incised by a blade (a) and a power blur then was used to thin maxilla and frontal process of the maxilla (b), a rongeur to remove the bone (c), and a probe to bulge medial sac and allow the medial wall of the sac fully incised (d); finally the entire sac was opened (e) and the wound was packed with merogel (f).

**Figure 2:**
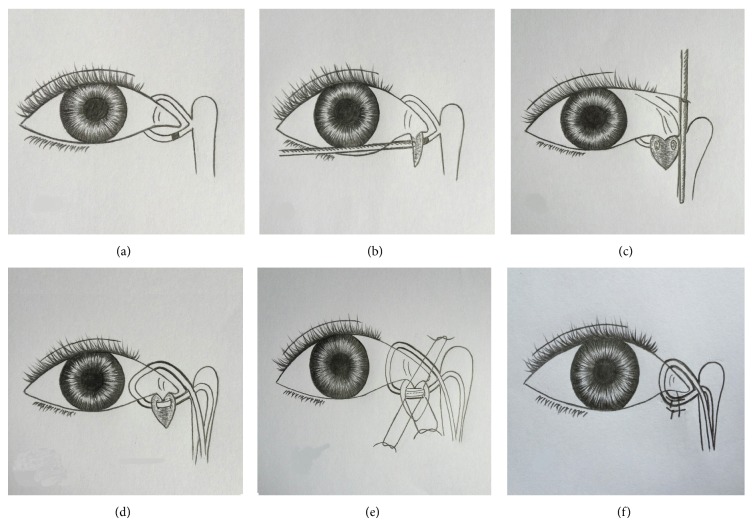
Canalicular repair. A lower canalicular obstruction is shown in (a). A probe is inserted to the obstructed site, and a vertical incision about 5 mm is performed according to the site (b); after inserting a probe from upper canalicular punctum to expose the distal end on a vertical axis of the probe, a pair of scissors is used to cut out scar tissue at the distal canalicular end (c); bicanalicular silicon tube intubation is performed through the reopened canaliculus into nasal cavity (d); 2-3 pairs of sutures are placed around the two ends of canalicular lumen (e); finally, the skin wound is closed with 2-3 stitches (f).

Four patients with bilateral dacryocystitis underwent their surgeries under general anesthesia and the remaining eight patients with unilateral dacryocystitis under local anesthesia. EE-DCR was performed firstly: A square mucosal flap above 8–10 mm to the operculum of middle turbinate was incised by a blade (Figure 1(a)). Underneath, the maxilla and frontal process of the maxilla were thinned by a power burr (Figure 1(b)) and then removed by a Kerrison rongeur (Figure 1(c)), to expose the entire medial wall of the lacrimal sac. Inserting a probe from the upper punctum to bulge medial sac (Figure 1(d)) allows a curved scleral knife to fully open the sac (Figure 1(e)). After checking the patency with saline irrigation via the upper and lower canalicular puncta, the nasal mucosal flap was trimmed and repositioned to cover the exposed maxilla and then packed with Merogel around the wound (Figure 1(f)). Next, the canalicular obstruction was repaired as the following steps: the site of a canalicular obstruction (Figure 2(a)) was detected by a probe under the aide of a microscope and a vertical incision around 5 mm was made according to the block site (Figure 2(b)). The scar tissue over the canalicular duct was carefully removed with scissors until the probe can freely slide into the reopened distal canalicular duct, which was identified on a vertical line to the probe that was inserted from upper punctum (Figure 2(c)). A silicon tube was inserted from the upper and lower puncta through the reopened canaliculus into nasal cavity (Figure 2(d)) and left the knot free in the nasal cavity [8, 9]. 2-3 pairs of 8-0 absorbable stiches were placed from the proximal end to the distal end around the canalicular lumen and tied them together (Figure 2(e)). The lid incision was finally closed with 2-3 stitches (Figure 2(f)).

Postoperatively, lacrimal syringing with dexamethasone and tobramycin was performed once daily for the first 3 postoperative days. Skin stitches were removed in 7 postoperative days. The clots and crusts in the nasal cavity were cleaned under a nasal endoscope in 2 weeks of postoperation. Follow-up period was set in 1, 2, and 4 weeks and 2, 3, and 6 months of postoperation, and then once a year. Silicon tube was removed around 3–6 months of postoperation. Slit lamp, fluorescein dye disappearance test, lacrimal syringing, lacrimal endoscopy, and nasal endoscopy are the major observations for each of the follow-up periods. The success rates were calculated according to the outcomes of EE-DCR and canalicular repair at the end of the follow-up. The functional success for the combination surgery was mainly defined as without epiphora and mucopurulent discharge and normal fluorescein dye disappearance test. The anatomical success was mainly defined as patent irrigation or probing.

## 3. Results

**Figure 3:**
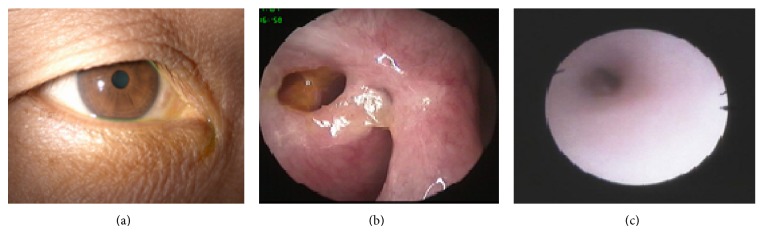
Postoperative photographs: (a) shows no remarkable skin scar presented at the site of lower lid incision; (b) shows an intranasal ostium widely opened, with fluorescein dye appearing around the intranasal ostium; (c) shows the smooth lumen when a lacrimal endoscope is used to examine the repaired lower canaliculus.

All 12 patients had a successful surgery in around 2 hours of surgical time. The complications associated with EE-DCR and canalicular repair included small granuloma around the intranasal ostium in 1 case and accident extrusion of the silicone tube in 1 case in 2 months of postoperation, but those complications did not result in reobstruction over the lacrimal system. No constantly bleeding or remarkable scar occurred (Figure 3(a)). After more than 6 months of postoperative follow-up, mucopurulent discharge disappeared completely in 12 patients, and all of them had a normal fluorescein dye disappearance test and patent intranasal ostium (Table 2, Figure 3(b)). The symptom of epiphora was completely disappeared in 10 patients, and the remaining two patients had occasional or intermittent epiphora (Table 2). Patent bicanalicular irrigation was achieved in 9 patients, and the remaining one had partial and the other two had a complete reobstruction in their repaired lower canaliculi (Table 2); however, all the three of them had a patent upper canaliculus. Those with patent lower canalicular irrigation showed a smooth lower canalicular lumen through the lacrimal endoscopic examination (Figure 3(c)). From the above results, 83% (10/12) of functional success rate and 75% of anatomical success rate have been obtained for the combination surgery. The outcomes are summarized in Table 2.

## 4. Discussion

It is well known that Ex-DCR or EE-DCR has over a 90% of high success rate for the management of dacryocystitis [4, 10]. EE-DCR has showed a number of advantages over Ex-DCR, including mini-invasive, preservation of medial canthal tendon and pump function, direct visualization of nasal anatomy, and avoidance of cutaneous scar [10–12]. In our case series, we used EE-DCR to manage 12 patients (16 eyes) with dacryocystitis and achieved a 100% of success rate. The success presented no mucopurulent discharge, patent irrigation, and opened intranasal ostium. Two patients (cases #1 and #11) with occasional or intermittent epiphora are not due to the failure of EE-DCR and likely due to the failed lower canalicular repair. It proves again that EE-DCR is a highly successful surgery for dacryocystitis. Canalicular obstruction is a less common lacrimal system disorder. A report from a meta-analysis showed the incidence of canalicular obstruction, nasolacrimal duct obstruction, and chronic dacryocystitis being 17.9%, 43.6%, and 18.4%, respectively [13]. However, canalicular obstruction has a worse outcome and prognosis than the other two lacrimal disorders, with an average 50%–60% of success rate [6, 7, 13–16]. The lower success rate is probably related to the long small canaliculus easily to be reinjured or reobstructed after surgical intervention. Until now, literature search has not found any reports mentioned about the incidence of the dacryocystitis combined with canalicular obstruction. Dr. Dalgleish reported a 10% (21/210) of nasolacrimal duct obstruction combined with canalicular obstruction but not mentioned dacryocystitis with canalicular obstruction [17]. We reviewed 900 series cases of EE-DCR performed in our hospital over past two years and found that 36 cases (4%) were dacryocystitis with canalicular obstruction. The most of them were with lower canalicular obstruction, for example, all cases in our study as lower canalicular obstruction. The reason for the higher incidence of lower canalicular obstruction may be due to the lower canaliculus as the major functional duct, in which the debris or inflammation is more likely started from the canaliculus. The other reason may be related to the widely used irrigation, probing, laser, or silicon tube intubation to manage lacrimal system disorder. Improper manipulations may induce injury especially in lower canaliculus; for example, lower canalicular obstruction may happen after probing to treat nasolacrimal duct obstruction. Since the upper canalicular duct is relatively less important during the drainage of tear flow, which counts about 30% of drainage efficiency comparing with about 70% of that in lower canaliculus [18], in normal condition, tearing may not occur when only upper canalicular duct is obstructed or inefficiently worked. Thus sometimes in clinic, when dacryocystitis is accompanied with upper canalicular obstruction, we might just perform EE-DCR and let the upper canalicular obstruction retain. However, when with lower canalicular duct obstruction, we need to consider repairing the lower canaliculus. In our twelve patients, all the involvement in canaliculus is lower canaliculus so we have to repair those obstructed ducts. Conventional treatment for canalicular duct obstruction mainly includes laser or intubation or both [6, 13, 16]. The other treatment method implicated for canalicular obstruction is DCR combined with trephination or membranectomy [9, 14, 19, 20]. Until now, optimized surgical method for the canalicular obstruction still has not been found. Although laser is increasingly used to break a scar tissue and reopen the obstructed canaliculus, and over three months of silicon tube intubation can enhance the success rate, narrow or reobstruction following laser or silicon tube removal could happen again. Moreover, when a canaliculus enters to the sac in a sharp angular, laser might not be easy to go through or might create a false passage. Under the situation without laser equipment, or very thick canalicular scar, laser or intubation could not be performed. In particular cases when upper and lower canaliculi are severely involved, Jones bypass glass tube has to be considered to be inserted [21, 22], otherwise, repair of canalicular obstruction has to be given up. Jones bypass glass reportedly has a good success rate; however, variety of uncomfortable complications may affect surgeon's decision to perform this procedure [21–23]. With less experience or rare case reports, the management of dacryocystitis with canalicular obstruction is rather difficult or complicated. Up to date, we also have not found any clinical study about using EE-DCR and canalicular repair to manage the complicated disorder. During the clinical practice, we combined EE-DCR with a modified lower canalicular repair to reopen the obstructed canaliculus and achieved a significant outcome. Since EE-DCR has a very good outcome for dacryocystitis, the key point for the management of dacryocystitis with canalicular obstruction is how to manage canalicular obstruction. The surgical technique we used for canalicular repair is modified from the traumatic canalicular repair [24, 25], including identification of the obstructed site, removal of the scar tissue, insertion of a silicon tube, and wound suturing. Some studies have demonstrated that the treatment of canalicular obstructions varies according to the level and extent of the obstruction [10, 14, 25]: if the obstruction of the canaliculus extends too far from the punctum to recreate the normal anatomical pathway, the canalicular system must be bypassed with conjunctivodacryocystorhinostomy and a Jones tube insertion. If canalicular obstruction is localized to the common canaliculus, laser-assisted canaliculodacryocystorhinostomy can be performed with silicone tube intubation. For the treatment of distal and common canalicular obstruction, a canaliculoplasty with trephination or internal membranectomy can be performed during dacryocystorhinostomy. However, those principles are a little complicated and need some special equipment. The modified surgery that we used can manage varieties of canalicular obstructions just simply using a microscope and a pair of scissors, whether the obstructed site is far or near from the punctum, or whether the scar tissue is thin or thick. Since the major obstruction sites locate at distal canaliculi, for example, in our series, over 5 mm in ten cases and equal to or less than 5 mm only 2 cases, how to find the distal lumen to allow the silicon tube insertion is the key step during the operation. The distal end within 5 mm is not difficult to find. For the distal end above 5 mm, after scar removal, the tiny tube could be buried in the lid tissue sometimes hardly to be found. In this difficult situation, we insert a probe from the upper canaliculus into the nose, in which the free distal end should be on the vertical line to the probe (Figure 2(c)). We did use this technique to successfully identify the distal end in all the difficult cases. It is also important to precisely remove canalicular scar in order to expose the normal lumen under a microscope and carefully anastomose two ends of a canalicular duct with 2-3 pairs of sutures. With a follow-up of 6–18 months of postoperation, being free of epiphora and mucopurulent discharge have been achieved in ten cases and one case with occasional and the other one with intermittent epiphora. The functional success rate for the combination surgery is 83% (10/12). We used lacrimal syringing, probing, and lacrimal endoscopy to examine the repaired canaliculi and found that nine of the canalicular ducts were completely opened (Figure 3(c) and Table 2). Remaining one canaliculus was with partial reobstruction and the other two were with complete reobstruction. Interestedly, the patient (case #4) with complete reobstruction in her repaired lower canaliculus did not have any epiphora or mucopurulent discharge, which may implicate a successful EE-DCR and compensative upper canaliculus obtained. The anatomical success of canalicular repair is 75%. Thus, the functional satisfaction (83%) is beyond the anatomical success (75%). Overall, the advantages of our combination surgery are, firstly, complete removal of the scar tissue over the canaliculus. Whether laser or silicon tube intubation is performed, the scar tissue is retained in the canaliculus. This method can be used in varieties of canalicular obstruction including easier or difficult case, especially when without the equipment like laser. Secondly, this method would not make a false passage, since we manipulate this procedure directly under a microscope, unlike a laser releasing a shot blindly. It is simple, safe, and efficient. It may become a good option for the management of dacryocystitis with canalicular obstruction. However, this study has some shortages, for example, limited cases, no control, and short-term follow-up. Next, we should add a control, recruit more cases, and follow up longer, to prove its efficiency and safety.

## Figures and Tables

**Table 1:** The information of 12 patients with dacryocystitis and canalicular obstruction.

No	Age (y)	Sex	Duration (y)	Dacryocystitis	LCO	Site of LCO (mm)
1	41	F	4	Unilateral	Left	6.3
2	77	F	2	Bilateral	Left	7.0
3	54	F	3	Unilateral	Left	6.8
4	65	F	8	Bilateral	Left	7.5
5	49	F	1	Unilateral	Right	3.0
6	31	F	5	Bilateral	Right	6.5
7	52	F	5	Unilateral	Left	7.0
8	59	F	20	Unilateral	Left	5.0
9	49	F	2	Unilateral	Right	6.7
10	53	F	20	Bilateral	Left	7.1
11	39	F	10	Unilateral	Left	6.7
12	37	F	5	Unilateral	Left	7.2

No: case number; y: years; F: female; LCO: lower canalicular obstruction.

**Table 2:** The surgical outcomes in 12 patients with dacryocystitis and canalicular obstruction.

No	Operations	Epiphora	FDDT	Irrigation (LCD)	Follow-up (m)
1	UEEDCR + LLCR	Occasional	+	Partial block	18
2	BEEDCR + LLCR	No	+	Patent	12
3	UEEDCR + LLCR	No	+	Patent	6
4	BEEDCR + LLCR	No	+	Complete block	9
5	UEEDCR + RLCR	No	+	Patent	10
6	BEEDCR + RLCR	No	+	Patent	10
7	UEEDCR + LLCR	No	+	Patent	7.5
8	UEEDCR + LLCR	No	+	Patent	7
9	UEEDCR + LLCR	No	+	Patent	6
10	UEEDCR + LLCR	No	+	Patent	8
11	UEEDCR + LLCR	Intermittent	+	Complete block	8
12	UEEDCR + LLCR	No	+	Patent	7

No: case number; UEEDCR: unilateral EE-DCR; LLCR: left lower canalicular repair; BEEDCR: bilateral EE-DCR; RLCR: right lower canalicular repair; FDDT: fluorescein dye disappearance test; LCD: lower canalicular duct; m: months.
